# Evaluation of a pilot to introduce simulated learning activities to support speech and language therapy students’ clinical development

**DOI:** 10.1111/1460-6984.12953

**Published:** 2023-09-16

**Authors:** Emma Ormerod, Claire Mitchell

**Affiliations:** ^1^ DPCHN, SHS The University of Manchester Manchester UK

**Keywords:** clinical competence, education, qualitative, simulation, speech and language therapists

## Abstract

**Background:**

Speech and language therapy (SLT) education must meet the needs of the future workforce, training enough students who are competent practitioners able to meet the workforce demands. Increasing student numbers and the impact on placement providers mean students must be equipped for learning on placement. Simulation is a way of supporting students to develop their clinical skills and decision‐making in a safe, supportive environment.

**Aims:**

To explore the perspectives of SLT students who were introduced to simulation during their undergraduate degree at a UK university as part of a pilot study. The aim of the pilot was to listen to the students’ voices to begin to understand their lived experiences of simulation and to gather views on how simulation can support their clinical learning.

**Methods & Procedures:**

Focus groups and semi‐structured interviews were carried out with second‐year BSc SLT students in semester 2 following the simulated learning activities and clinical placement. Qualitative data were gathered and thematic analysis was applied to the data to identify the barriers and enablers to students’ clinical learning in simulation.

**Outcomes & Results:**

A total of 11 students responded out of a cohort of 38. Three key themes were identified from the analysis: individual learning needs, facilitator skill and programme‐level organization.

**Conclusions & Implications:**

Student experience of simulation was positive. One of the key elements students found to support their clinical skills was the importance of the safe space; support for learning instead of correction led them to engage in active learning. Key barriers to simulation related to having sufficient prior knowledge, the skills of the facilitator, group size and the wider learning landscape of the programme. In response to this pilot, there are plans to continue developing this model of simulation and embed simulation across the programme, led by a sound pedagogical approach with clear preparation and planning and building the necessary infrastructure. Other SLT programmes and practice educators developing simulation as part of their programmes or placement may wish to consider some of these findings to support the use of simulation in their workplace.

**What this paper adds:**

**What is already known on this subject**

- Simulation as a teaching methodology is widely used in medicine and nursing programmes. It is now used in various allied health professions and in some SLT programmes. There is evidence to suggest simulation increases student confidence and clinical skills without increasing the capacity on those offering clinical placements in practice.

**What is already known on this subject**

- This study offers a practical example of introducing simulation in an established undergraduate programme for SLT students. It explains the background to this innovative way of teaching clinical skills and explains why this approach could be beneficial for the future speech and language therapist.

**What are the clinical implications of this work?**

- This study gives practical examples of how simulation can work to facilitate student clinical learning and knowledge. It may offer ideas to those working in clinical practice to organize placements differently or add simulation elements to improve the student experience. Other educational establishments and placement educators may find the recommendations helpful in developing their own simulation approach.

## INTRODUCTION

Speech and language therapy (SLT) training programmes at degree level have changed over the years as the profession has evolved and SLT educators have responded to the emerging, scientific evidence base for both the profession and pedagogical evidence. Programmes must also continue to adapt how they develop their trainee health professionals to meet the changing needs of the population, most recently in response to the strategic vision for care as set out in the National Health Service's (NHS) long‐term plan (NHS England, 2019). To be able to deliver the agenda set out to address prevention, integration of community services, reduction in unplanned emergency admissions and reducing health equality, we must ensure education of the future SLT workforce is fit for purpose. The training needs of all allied health professions, which include speech and language therapists, must focus on collaborative, multi‐professional working as described in the NHS people plan (Bailey, 2020). Allied health professionals have long been considered a key part of the workforce, ideally placed and trained to support innovation across the NHS to transform health, care and well‐being, and it is crucial that speech and language therapists remain at the forefront of innovation (England, 2017).

To be able to deliver the NHS's long‐term plan, we need to have sufficient numbers of speech and language therapists trained to deliver care in this way. There is already a shortage of speech and language therapists in many clinical areas, and specifically at certain grades (Buchan et al., 2019). Higher education institutes are setting up new SLT programmes and expanding existing programmes, with a variety of routes to professional qualification. This will inevitably place additional pressure on those delivering clinical placement with greater numbers of students requiring placements. It may therefore be prudent to consider the wider clinical learning environments available and consider how these can support clinical learning. Students must be well‐prepared to make the most of their time on clinical placement to ensure they achieve clinical competence across the wide range of complex, clinical situations in which SLTs work (Quigley et al., 2020). One way to achieve on‐campus clinical experience and better prepare students for placement is through simulation. Simulation is a ‘technique’ or teaching methodology that allows students to be immersed in a replicated ‘real life’ situation to develop clinical skills (Aiken et al., 2003; Gaba, 2007; Hewat et al., 2020).

Simulation has been used for many years across healthcare, particularly in nursing and medicine, with a robust evidence base (Gaba, 2007; Hamada et al., 2020). There is a growing use of healthcare simulation across allied health professional training programmes (Astbury et al., 2021) and increasing research into the benefits of this teaching methodology (Alanazi et al., 2017). Programmes around the world are using simulation in SLT, with research from Australia suggesting it can replace clinical placement hours to achieve the same competencies (Hill et al., 2021). Simulation can offer a wider variety of clinical experiences than may be possible on clinical placement. There is evidence that simulation can offer students parity of experience where placement can be hugely varied, and allow them to experience more challenging or complex situations that they may be protected from by their placement educator (Siew et al., 2021). The simulation literature talks about fidelity, realism and levels of immersion as some of its key benefits. Lioce et al. (2015) discuss the level of immersion required and how ‘real’ the simulation feels as key considerations in planning and implementing simulation. Levels of immersion can vary depending on the setting and context. This can range from being in a realistic simulated clinical environment (i.e., a hospital ward or patients home), or it could include generic rooms with students encouraged to consider themselves in a particular clinical environment. It can also include simulated patients (i.e., a simulated patient and/or standardized patient refers to someone who takes on the role profile of, for example, a patient, family or healthcare roles), video footage or paper‐based scenarios or students role‐playing scenarios (Hill et al., 2010).

This teaching methodology involves preparation of the students before the simulation which sets up a supportive environment in a safe learning space where students can test out their clinical skills, pause and reflect (Rudolph et al., 2014). Simulation is often delivered in small groups to allow peer learning and support by a trained facilitator who supports the clinical learning experience. Simulation has been reported to improve attitudes and confidence, which can support multidisciplinary teamwork (Hamada et al., 2020; Jakobsen et al., 2018). In addition, simulation is a way of providing clinical learning that offers clinical experience without being on placement and does not involve working with actual health and social care users (Hill et al., 2021).

Given its above noted benefits, simulation was introduced to second‐year students on the University of Manchester's undergraduate SLT degree programme. The simulation scenarios were designed to introduce clinical and professional communication skills and develop clinical reasoning through detailed scenarios. The pilot study aimed to explore SLT students’ perspective of simulation. It also aimed to understand students lived experience of simulation and to gather their views on how simulation can support clinical learning.

## METHODS

### Intervention

During one academic year, teaching methods on two, second‐year course units were updated to include a mixed‐model approach of simulation supported with preparation workshops and independent online learning units. This was a change from previous lectures.

When developing the simulations, it was important to achieve authenticity within a limited budget. A range of resources were used, including videos of real patients. Local SLT services were approached to identify clients who would be appropriate to meet the learning objectives for the proposed simulations and were willing to consent to being filmed for simulated learning activities. In order to replicate a clinical observation, the client was videoed from the perspective of a student observing in the clinic room. This allowed students to observe real clinical sessions, including assessments and clinical interventions, and real clients without having to travel to the clinical setting.

The short series of simulations, running over consecutive weeks, mirrored the clinical process, including observation of assessment, recording of information, case note writing and analysing data. Members of the wider teaching team took on the role of simulated patients, including the role of parent or family member in order to give students experience of feeding back information and using their clinical communication skills. Using members of the teaching team did lower fidelity given students were familiar with these staff members. However, as an introduction and orientation to clinical skills and clinical reasoning skills, the use of teaching staff within the simulation achieved an appropriate level of fidelity within a limited budget and offered an opportunity to explore a range of simulations.

Inpatient simulations were delivered in the clinical skills suite/simulated inpatient ward and all other simulations were delivered in flat rooms for small group work. Simulation sessions included a range of clinical cases and content in order to meet designated learning objectives. Table 1 details cases and student learning activities.

**TABLE 1:** Simulated cases and student learning activities.

Case presentation	Student learning activities
Inpatient dysphagia clinical case: acute stroke	Clinical swallow examination
	Video fluoroscopy (VFL) workshop and sharing findings from VFL assessment
	Feeding back dysphagia recommendations to a family member
Dysphagia outpatient clinical case: (chronic obstructive pulmonary disease (COPD)	Information‐gathering and case history‐taking
Paediatric clinical case	Initial assessment
School‐aged child with language difficulties	Analysing the assessment findings
	Planning therapy
	Feeding back to a parent
Adult acquired clinical case: stroke	Goal‐setting
	Assessment
	Intervention‐planning

Following each simulation, all students took part in a verbal group debrief. This included debriefing the scenario and debriefing the students’ learning. There are numerous debrief models used in simulation, including the diamond debrief model (Jaye et al., 2015) and debriefing with good judgement (Rudolph et al., 2014). Given the intended learning outcomes of these simulations, a version of ‘stop, start, continue’ (Hoon et al., 2015) was deemed to be the most appropriate. In this debriefing mode, students are encouraged to share one thing they want to stop doing, one thing they want to start doing and one thing they want to continue doing.

### Design

This evaluation study involved individual interviews and focus groups using semi‐structured interview techniques to generate students’ views of their experience of simulation.

The interview schedule consisted of open‐ended questions that allowed the students to share their perspective on the wider benefits of simulation, what it offered them as individuals, how it prepared them for placement and the challenges to learning in this way. Students were encouraged to share their views of reflection, the use of videos and the role of the facilitator as well as practical elements such as rooms and relationship to the wider programme of education and clinical learning (see Appendix A). Focus groups offer a way for participants to give their honest and spontaneous opinions, ideas and perceptions in a dynamic way providing a detailed narrative for in depth analysis. Focus groups also provide insights into the co‐constructed opinions of members of the same group (Barbour, 2018).

### Participants and data collection

Student participants were recruited from the University of Manchester undergraduate SLT programme. All were second‐year students who had experienced simulation. Emails were sent to the second‐year cohort (sample size of 38) and the study was discussed in face‐to‐face sessions. The focus groups were set up to accommodate student attendance, avoiding placement and teaching sessions and at times convenient to them. An individual interview was conducted with one student to accommodate their availability. Two focus group meetings and the individual interview (*n* = 11 students in total) were held on different days and times. One focus group and the interview were carried out by one facilitator (lead author), and one focus group was carried out by another facilitator (co‐author) on campus. We attempted to mitigate bias by ensuring that educators interviewing the students or carrying out focus groups had not directly taught the students. Students were encouraged to speak freely without concern that their honest opinions would affect their progression on the programme. The individual interviews and focus groups lasted between 1 and 2 h.

Informed consent was obtained from all study participants and formal ethics approval was gained from the University Ethics Board (2019‐6022‐9895).

### Data analysis

The focus groups and individual interviews were audio recorded with written consent and transcribed verbatim and anonymized before being transferred to NVivo 11 software to store and manage the data. Data analysis was approached thematically, using an inductive analysis approach (Braun & Clarke, 2006). A coding framework was developed by the two authors E.O. and C.M. through interpretation of the data and the related literature, focusing on barriers and enablers to the student view of simulation (King, 2012). Further codes were added to the framework inductively, as appropriate, and then coded across all transcripts. Once all transcripts were coded, the findings relating the student views were in addition compared across the focus groups and individual interview to identify similarities and differences across them.

The following key of FG = focus group and SSI = semi structured interview with participant letter from A to K was applied.

## RESULTS

The aim of this pilot was to explore SLT students’ perspective of simulation. It also aimed to understand students’ lived experience of simulation and to gather their views on how simulation can support clinical learning. Three themes emerged from our thematic analysis: (1) individual learning, (2) the role of the facilitator and (3) organization and delivery (programme level).

### Individual learning

A key enabler from this study was that students found the simulated learning environment to be a safe space where they felt supported and able to make mistakes. They understood that this was an opportunity to practise and prepare in a live immersive situation but without the risks of harm associated with an actual placement with patients:

I think role play is really good. In the paediatric one I remember I was doing a role play interviewing a parent. I was using all of these big words. The clinical educator was pretending to be the parent and asking what does that mean. I was kind of like I do not actually know how to explain that. It was really useful to be doing the role play and asking for a pause … then jump back in. better to pause than struggling through role play, less stressful. (FG I)

This type of simulated case allowed students to experience complex clinical situations and consider their existing knowledge and what other information they need to find out. This allowed them to understand their own levels of competence and what support they may need:

For me it was pinning down a lot of theoretical learning. It was saying ‘right, now you have got all that, where is it actually taking you? What are you actually going to do with that? We spend a lot of time understanding what assessment we are going to do but we do not think about what therapy we actually do. (SSI)

Students valued the opportunity to be able to make mistakes without judgement or assessment and to reflect, repair and learn from these:

It is a safe environment … because it is not assessed or something like that you just feel free to make mistakes and to learn in that way. It just feels like a really supportive environment kind of to explore all these new things. (FG G)

Students reflected on the fact they have to engage in the session and to come up with ideas and a plan for therapy as they would in a ‘live’ placement environment. Student H commented that simulation differs to a lecture in that debrief and reflection made them more active learners.

What I really did like at the end having that chance to think about what you have learnt, what I had changed, expected. I liked the whole process. It makes you think it is very easy to walk out of a two‐hour lecture and think ‘what are we doing now’. It did make me stop and think actually, what would I change? what would I do differently? (FG H)

Some of the barriers to simulated learning from the student perspective related to the level of debrief received and the lack of individual feedback about their clinical skills. A small number of students suggested they would like to receive individual personalized feedback on their clinical skills in the safe simulation environment rather than on practice placement only. As well as debriefing their individual learning students also wanted to debrief the scenario to know if they were ‘right’ or ‘wrong’ in their clinical decision‐making. Students sometimes struggled with the complex decision‐making that does not always have a clear right or wrong answer.

I think more personal feedback rather than just general feedback. On the first session, it could be you did well but you could have improved, or what you understand to improve. You get personal feedback on exams and assignments but not so much on how you are clinically. (FG I)

### Facilitator skill

Students reported they had a different relationship with their lecturers working with them in simulation.

You are interacting more and that is the sort of thing you get on placement. (FG G)

There was a sense that the facilitators needed to allow the students the chance to work through the clinical situation and they did not need to be guided the whole way.

I like it when they let us figure it out for ourselves and then to get the answer at the end/ the debrief. Having that discussion and working to come to an answer between the group but need the debrief at the end. Gives independence rather than being talked at (FG H).

The tone of the simulated learning environment was important. Students responded well to strong, well‐facilitated sessions that focused on building on existing knowledge and encouraging all students to be actively involved.

It is a difficult skill to have, but basically, not saying that is wrong. It was a nice environment and people I know from that group who do not comment in lectures were commenting, and I think that is helpful as well. (SSI)

The students commented on different styles of facilitators, which is a challenge to implementing simulation. Facilitators have their own ways of working and how they facilitate the sessions can have an influence on the direction and learning outcomes. Students commented that the most effective learning from facilitators happened when they guided students in their thinking rather than providing an expert opinion

Sometimes it is clear they are delivering a session on something they know a lot about, they are specialist in or not and sometime they are not a specialist in and that come across in the delivery. Sometimes the clinical educator may speak more if wanting to teach us something and sometimes we are left more to think on our own and figure it out. (FG K)

### Programme level organization

Students commented on the importance of the surroundings to set the scene for the simulation and support the immersive experience. The inpatient dysphagia simulations were in a clinical skills suite on a simulated inpatient ward, which helped students to immerse themselves in the session. Students were clear that being in a traditional lecture theatre would not be conducive to simulated clinical learning sessions and would be distracting:

really useful being in the simulated clinic you know with the beds … with the beds it felt more like you were in a placement setting rather than sat in a lecture theatre or something like that. … It helps you to apply the knowledge you get in to a different setting and your role in that setting. (FG H)

There was some discussion about the levels of realism. The use of videos of real cases helped to personalize the case and orientate students to the client. The use of simulated patients allowed for a more immersive learning experience:

Both are good. Videos to get your first initial ‘oh that is what that is …’, but when you see something real like when people do role play … and I'd be like oh I never would have thought of saying that. But when you are watching a video you are not focussing as much because it is a video when it is real people you interact more. (FG D)

Group size was also an important factor. The facilitator to student ratio was seen as an enabler to allow more students to engage and discuss. Some student groups felt t they did not get enough facilitator time; this may be due to the various sizes of groups that were trialled:

People are far more likely to interact during those sessions. In lecture theatres and lecturer asks a question there is a big silence. And no one speaks but when there is a group of 8 and you go round and all say stuff. I think the group size either makes or breaks it. There have been sessions where it has been the entire class, and that has not worked at all, but this one where there were just eight of us that is the ideal size, and I think it works if the group size is manageably small. (FG K)

The students commented on the importance of seeing clinical learning across the programme rather than in separate strands. They found the linking of theory to practice during the semester as an important way to keep their clinical practice and skills relevant and relatable to theory:

When you are on placement learn so much and then when you think you need to go back to university it feels like 2 completely different things. These sessions remind you they are not two different things. They remind you to keep applying things and it gives you more motivation. (FG J)

Students discussed how simulated clinical sessions could support placement and prepare them better for their placements. There was a sense from students that simulated learning activities helped them to practice and prepare ahead of placement and start to work on some of their clinical competencies:

If you had that before a placement, it would be a massive increase in confidence. I really cannot state that enough, the way you think about or the way you tackle something, the process, how you go from referral to discharge. Yes, a huge impact. (SSI)

I think as well having a few opportunities to do the role‐play throughout the course. Because if I think back to the first one—I really think it helps to develop your interpersonal skills, and everyone is so varied and if you did not have an experience of adults or children, it is a good way to develop that as well. (FG J)

Students highlighted the importance of having opportunities to practise and develop their interpersonal skills that underpin clinical communication skills. Interpersonal skills can be challenging to teach in a lecture setting. Simulation offers a way to explore and practise human factors in an experiential and psychologically safe way.

I think it is very difficult to teach someone how to be compassionate, I think that has to be active practise, you cannot give a lecture on compassion, or conflict management. (SSI)

Some of the barriers around simulation that the students reported was how it fit with their theoretical learning.

In order to create space in the timetable dysphagia teaching included taught lectures and e‐learning for students to access independently alongside the simulations. As this was a new subject area for most, students indicated they would prefer to have lectures first and then simulations to consolidate and synthesize learning.

I think lectures first then simulation would be a little bit more beneficial because then you have got an idea of what you can do in the simulation or like do a couple of lectures then simulate it. That way you can say I struggled with this and then you have another lecture to back up what you have done, I do not know. (FG B)

For the adult acquired one, we had the simulation but because the lecture content was on ispring, I felt I could not ask the questions that I wanted to ask, and I did not know where to ask them because we did not have lots of lecture time. I ask lots of questions that is how I learn, and then I went to ask something and they said there was not much time to discuss. So I did not feel like I got the answer I wanted. (FG G)

Students raised concerns about parity of their experiences with simulated learning environments. They felt there was some disparity between the sessions, with different facilitators having differing approaches and feedback and differences in how the debrief was carried out, with a lack of debrief reported by some groups.

For me, for key themes to be highlighted for everyone, to make sure learning is same for everyone. (FG G)

## DISCUSSION

This evaluation of the introduction of simulation onto the University of Manchester's SLT programme was carried out to explore the perceptions of SLT students’ perceptions of simulation and to gather information of lived experiences to understand how simulation can support clinical learning. Carrying out focus groups and interviews enabled exploration of the barriers and facilitators to students’ learning and a way to understand the wider context of these simulated sessions within the existing programme. The focus groups and interviews supported students to express, honestly and clearly, what they thought did and did not support their learning. As part of this process, they offered their own thoughts, insights and suggestions for ways to improve the use of simulation. The three key themes that we identified from the student data were; individual learning needs, facilitator skill and programme‐level organization. This evaluation focuses on a SLT undergraduate degree programme but has the potential to offer recommendations to other allied health professional programmes who are currently using or wanting to introduce simulation.

There was unanimous feedback from students that simulation is an effective teaching methodology. They particularly valued the safety of the simulated learning environment (Hewat et al., 2020).

### Programme‐level organisation

Developing the infrastructure and foundations is essential when introducing simulation. In the current evaluation study, this development was seen as insufficient. In particular, facilitator training was minimal and curriculum planning needed further development. Feedback from students suggests simulation works best when offered as part of a blended curriculum alongside various different teaching methodologies. This is consistent with Alinier (2007) who advocates for students to access a variety of learning environments and teaching methods in a way that matches the student's level of learning and experiences. Students value having access to a range of teaching methodologies and learning environments for them to acquire new information and learn new skills. Intended learning outcomes should be constructively aligned, to ensure the most appropriate teaching methodologies and assessments are used (Biggs, 2012).

From the pilot and initial data, students value the model of having taught lectures followed by simulations to help apply, consolidate and synthesize their learning. Opportunities for applying learning across the programme are also beneficial so students become familiar with this way of learning. Indeed, there are numerous studies in support of embedding simulation to help students gain their competency outcomes (Ferguson et al., 2020; Hill et al., 2010). This has led to changes in the curriculum on the BSc SLT programme at the University of Manchester to have a blend of lectures followed by simulations timetabled as part of protected clinical learning environment days across the semester. As simulation continues to develop within the programme, it is important to continue to align this type of learning alongside theoretical and clinical learning. This alignment supports students to have a variety of experiences and enables staff to use different methods of teaching to support students meeting their competencies via a range of practise‐based learning opportunities (Sheepway et al., 2014, 2011).

### Facilitator skill

Once the curriculum has been set, when using simulation it is essential staff are trained in this teaching methodology and familiar with the facilitator role. Strong facilitation is closely linked with the safe learning environment that allows students to develop their knowledge in a supportive way (Lateef, 2010) both in simulation and on placement (Davies et al., 2019). The key to promoting this psychologically safe space is the role of the facilitator and their skill in promoting this (Edmondson, 2018).

The importance of the facilitator role and the debrief are fundamental to the success of a simulation (Lioce et al., 2015). Due to time constraints with getting the simulations up and running, facilitator training was not prioritized sufficiently. Students highlighted this suggesting as this is a different way of learning and indeed a new way of teaching for some facilitators there needs to be detailed and robust facilitator training to achieve parity of experience.

The facilitator role is essential in building psychological safety. This includes role‐modelling and offering regular feedback and support so students know they are on the right track and feel supported to actively engage in and contribute to the simulations.

In some simulation programmes, there are timetabled slots to give individual one‐to‐one feedback. There is evidence to suggest in specific contexts that personalized feedback from the facilitator can aid the student's learning (Penman et al., 2021). This was not included in this simulation programme due to time constraints. A small number of students raised this as something that is missing and something they would want. However, with clearer feedback, facilitation and signposting during the session, students should feel confident they are engaged in the learning process. Having a clear pre‐brief and debrief and clear orientation to students will help clarify the type of feedback students are receiving.

### Individual learner need

A core theme around individual learner need was trust. Students wanted to take responsibility and be trusted to work through the case and to ask for help when needed. This replicates the real world and the autonomy of practicing speech and language therapists.

Another key finding was the benefit and value students placed on the safe learning environment afforded by simulation. The reframing of mistakes and failure and some discomfort as part of learning allowed students to feel safe to make and correct their mistakes in this safe environment. Indeed, Hewat et al (2020) found practising in this safe and supportive environment increases confidence and reduces anxiety.

In order for this to be effective, the levels of learning must be appropriate for the students to meet their intended learning outcomes. A benefit of simulation is that there is consistency and continuity (MacBean et al., 2013). Making sure scenarios are appropriate for the level of learning, helps students to gain confidence and for the facilitator to trust they will be able to manage the case/clinical scenario independently.

## CONCLUSIONS

The use of simulation in an undergraduate SLT degree programme was highly rated by students. To be of most benefit simulation should be embedded across a programme to provide ongoing opportunities to practice and prepare in this safe learning environment. Simulations need to be well facilitated and well planned with appropriate pre‐brief and debrief opportunities for students. In order to do this, the infrastructure to support simulation development and delivery across the BSc programme is needed. This includes investment in developing the learning landscape to support this type of experiential learning and robust training for facilitators.

Simulation offers a sound pedagogical way to acquire clinical and professional skills and competencies as part of a range of practise‐based learning opportunities (Hill et al., 2021).

Simulation has a lot to offer by providing a safe place to learn and parity of experience. The key underpinnings for planning simulation are consideration of the individual learner need, facilitator skill and embedding in the curriculum with appropriate learning landscape in which to deliver simulation.

## CONFLICT OF INTEREST STATEMENT

The authors report no conflicts of interest. The authors alone are responsible for the content and writing of the paper.

## Data Availability

The data that support the findings of this study are available from the corresponding author upon reasonable request.

## FOCUS GROUP QUESTIONS

Questions for semi structured interview:

1. How would you describe the case‐based simulation work?
2. What do you think might be the benefits of learning in this way?
3. What does simulation offer you in terms of preparing for placement?
4. Can you think of any challenges with this way of learning (i.e., simulation)?
5. How do you find the reflection part of simulation?
6. How useful are the videos of the patients in the simulations?
7. Do you have any thoughts about how the sessions were facilitated by the clinical educator?
8. What other factors do you think are important with simulation?
9. How important is the environment?
